# Domingo Sánchez y Sánchez (1860–1947): Cajal’s man on the nervous system of invertebrates

**DOI:** 10.3389/fnana.2023.1330452

**Published:** 2024-01-09

**Authors:** Adela Serrano-Herrera, Juan Manuel Espinosa-Sanchez

**Affiliations:** ^1^Department of Otolaryngology, Hospital Universitario Virgen de las Nieves, Granada, Spain; ^2^Instituto de Investigación Biosanitaria ibs.GRANADA, Granada, Spain; ^3^Otology and Neurotology Group CTS495, Department of Genomic Medicine, GENYO. Centre for Genomics and Oncological Research: Pfizer / University of Granada / Andalusian Regional Government, PTS, Granada, Spain; ^4^Department of Surgery, Division of Otolaryngology, University of Granada, Granada, Spain; ^5^Sensorineural Pathology Programme, Centro de Investigación Biomédica en Red en Enfermedades Raras, CIBERER, Madrid, Spain

**Keywords:** Domingo Sánchez, Cajal, neurohistology, neuroscience, invertebrata

## Abstract

Domingo Sánchez y Sánchez (1860–1947), a distinguished disciple of Santiago Ramón y Cajal, played a fundamental role in the Spanish School of Neurohistology through the meticulous use of diverse staining and microscopic techniques in the study of the histology and physiology of the invertebrate nervous system, generating valuable contributions that were recognized and cited by the scientific community. His research covered a wide range of areas: he was initially an anthropologist and zoologist, later earning a doctorate in Medicine and specializing in the neurohistology of invertebrates, including the detailed study of the retina and nerve centers of insects, and the discovery of histolysis in nerve centers of insect larvae during metamorphosis, challenging scientific paradigms of the time. Furthermore, Sánchez’s work on the neurofibrils of insects was crucial in supporting Cajal’s neuronal theory and refuting Bethe and Apathy’s reticularist hypothesis. Additionally, he also made preliminary observations of the Golgi apparatus, the lysosomal system, the endoplasmic reticulum, and the sarcoplasmic reticulum of skeletal muscles (Cajal-Fusari network). Domingo Sánchez y Sánchez’s exceptional scientific research and contributions to neurohistology in 20th century Spain continue to serve as a significant legacy.

**Life science identifiers:**

*Apis mellifera*: urn:lsid:zoobank.org:act:9082C709-6347-4768-A0DC-27DC44400CB2

*Helix aspersa*: urn:lsid:zoobank.org:act:9099927E-24DF-4F89-B352-6B7902CD4A38

## Introduction

The magnitude of Cajal’s figure and work has been documented in hundreds of publications, some of them truly magnificent (López Muñoz and Álamo, 2006). However, the heroic, epic, and even mythic dimensions that have sometimes been attributed to his figure have contributed to almost forgetting the lives and works of his disciples, overshadowing the school he founded, and this is a serious disservice to his memory. Initiatives such as the Frontiers in Neuroanatomy Research Topic article collections have helped to remedy this harm (de Castro and Merchán, 2016).

The Spanish Histological School emerged with the contributions of Aureliano Maestre de San Juan (1828–1890) and is characterized by two primary branches. One of these branches is the Cajal’s School, which is specifically devoted to the histological study of the nervous system. On the other hand, there is the so-called histopathological branch, in which successive figures such as Luis Simarro (1851–1921), Nicolás Achúcarro (1880–1918), and Pío del Río-Hortega (1882–1945) are intertwined. Undoubtedly, there existed reciprocal influences between these two branches. Río-Hortega was, after Cajal, perhaps the most prominent figure in the Spanish Neurohistological School. He invented the method of ammoniacal silver carbonate, which allowed him to discover microglia and oligodendroglia. He also studied the cytology of the pineal gland and excelled in the surgical pathology of central nervous system tumors (Río-Hortega, 2013). Among the direct disciples of Cajal, we include Francisco Tello, Pedro Ramón, Domingo Sánchez, Fernando de Castro and Rafael Lorente de Nó. All of them have received specific attention except for Domingo Sánchez.

Francisco Tello (1880–1958) was the first pupil and Cajal’s right-hand man. He investigated the embryology of the cerebellum, the degenerative and regenerative processes in nerve endings, motor plates, neuromuscular spindles of the skeletal muscles, the regenerative processes in the optic tracts, and the influence of neurotropism in the nervous centers. He was also the introducer of modern Surgical Pathology in Spain (Martínez-Tello, 2020). Pedro Ramón (1854–1951) was the younger brother of Santiago. He contributed to the development of the law of Dynamic polarization, with special attention to comparative neurohistology in lower vertebrates. He was also a full professor of Gynecology and Obstetrics (Ramón y Cajal and de Carlos, 2020). Fernando de Castro (1896–1967) and Rafael Lorente de Nó (1902–1990) played a leading role in the transition from neuroanatomy and neurohistology to neurophysiology within the Cajal’s school. The first of them studied the structure and function of sympathetic and sensory ganglia, ganglionic synapses, and in 1927, he described the chemoreceptors of the carotid glomus. In fact, he was the first author to attribute a chemoreceptive function to the carotid body (de Castro, 2016; Ros-Bernal and de Castro, 2020). Lorente de Nó was the last and youngest of Santiago Ramón y Cajal direct disciples. He excelled in the study of the cytoarchitecture and the functional organization of the cerebral cortex, the anatomy and physiology of the audiovestibular system, and the mechanisms of synaptic transmission (Larriva-Sahd, 2014; Espinosa-Sanchez et al., 2020, 2023).

**Figure 1:**
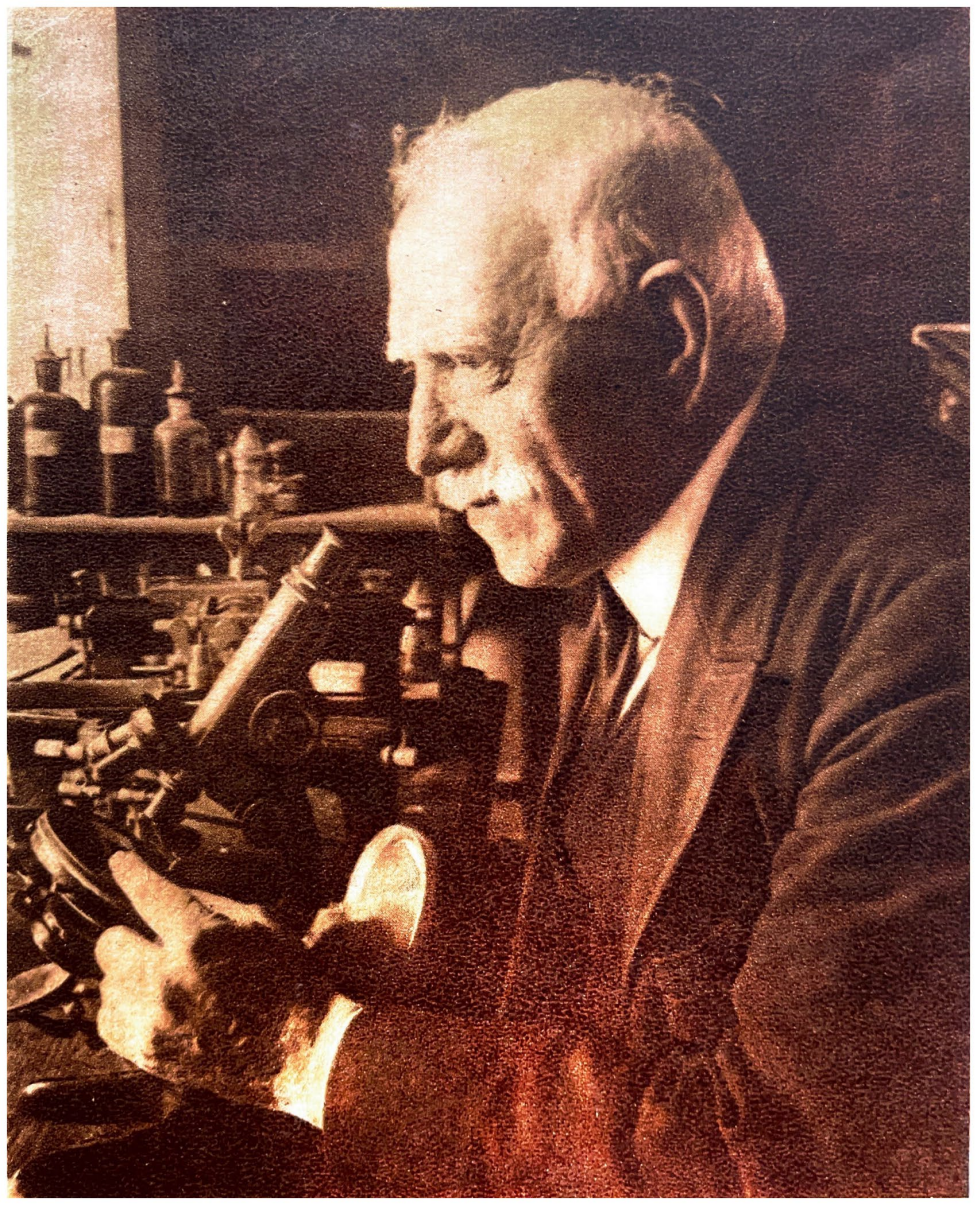
Photograph of Domingo Sánchez y Sánchez. ABC no extraordinario 21 July 1929. Madrid.

The scientific biography of Domingo Sánchez y Sánchez (1860–1947) is marked by a clear distinction between two crucial phases in his scientific career. This study examines the life and contributions of a scientist whose career spans from his work as a naturalist in the Philippines to his collaboration with the renowned Santiago Ramón y Cajal in Madrid, which prompted a prolific histological investigation of the microscopic structure of the invertebrate nervous system. His humbleness and affinity with his mentor, Cajal, influenced his role as one of the most prominent disciples of the Spanish Neurohistological School. This article analyzes his scientific career and its impact on the fields of histology and neuroscience in 20th century Spain (Figure 1).

## Biographical context

**Figure 2:**
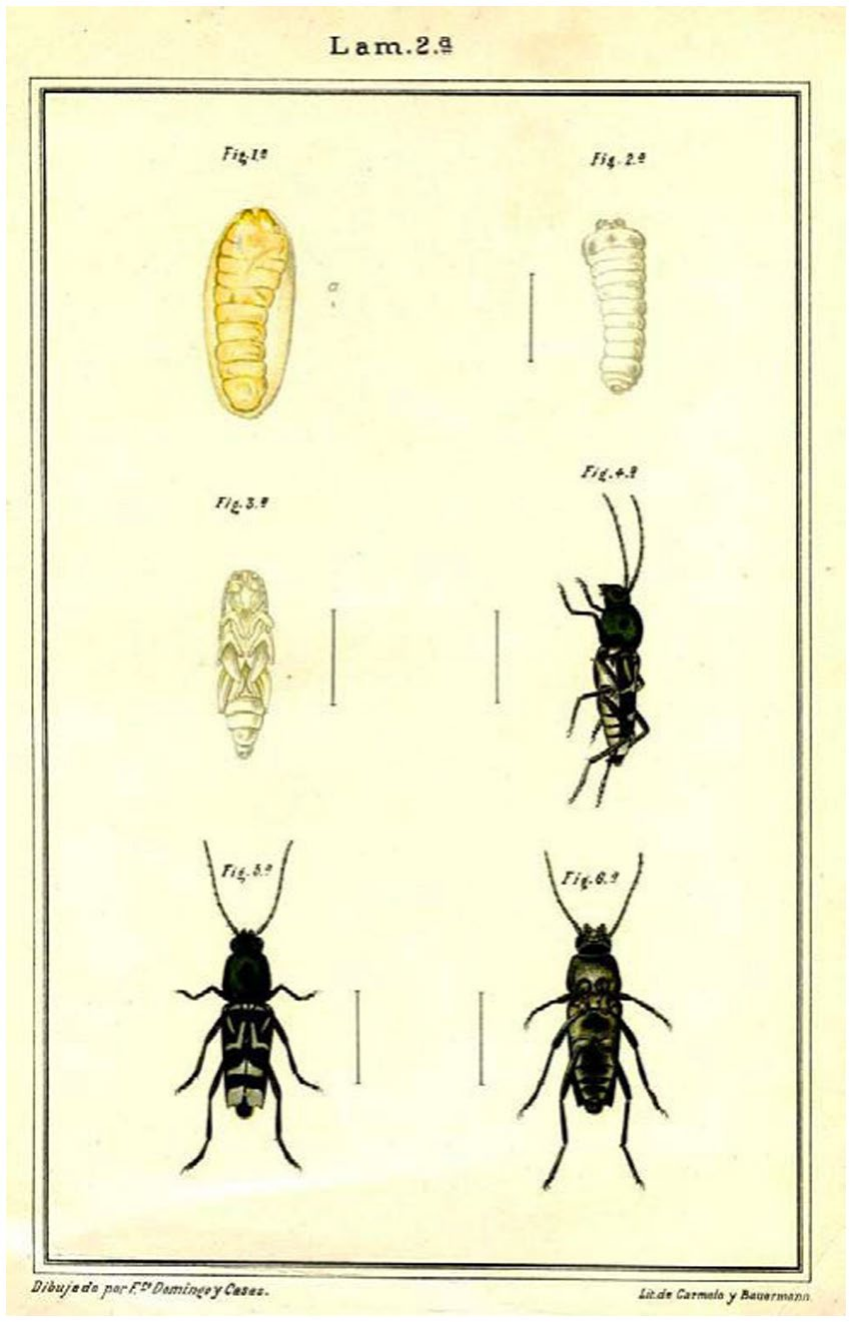
Insect enemy of coffee trees (Sánchez, 1890).

Born in 1860 to modest farmers in Fuenteguinaldo (Salamanca), he commenced his studies with the parish priest of his town at 15, having previously worked in agriculture and livestock farming. He subsequently enrolled in the seminary of Ciudad Rodrigo (Salamanca), pursuing his high school studies in the secondary schools of Salamanca and Avila. Following this, he completed his studies at the age of 21 and was awarded the extraordinary prize (Barras de Aragón, 1949). He went to Madrid to study natural sciences, which he finished in 1885 (Sánchez, 2019). In August of that year, he traveled by ship to the Philippines, then a part of Spanish Empire, as a zoological assistant to the Flora and Fauna Commission of the Philippines, having accepted the position on the condition that he would not travel until he had completed his studies (Sánchez, n.d.). In 1886, he was tasked with collecting, organizing, and categorizing zoological specimens for the General Exposition of the Philippines in Madrid in 1887. The following year, he returned to the peninsula and oversaw the installation and cataloging of the zoological material transported from the Philippines while simultaneously pursuing his doctorate. Upon the completion of the exhibition, he was appointed as an zoological assistant in the government forestry services (Barras de Aragón, 1949) and returned to the Philippines to investigate a disease that posed a risk to the coffee plantations (Sánchez, n.d.). Subsequently, he authored a report detailing the invasion of *Xylotrechus quadripes,* a type of long-horned beetle that inflicted a plague on coffee plantations in the Philippines (Sánchez, 1890; Figure 2). He spent nearly 14 years in the Philippines conducting expeditions across the archipelago, gathering specimens of flora and fauna, and also collecting anthropological material, which at times included looting mortuaries (Sánchez, 2019). While residing in Manila, he served as an active naturalist of the Provincial Board of Fisheries of Manila and also taught at the School of Arts and Crafts (Sánchez, n.d.).

In 1896, he returned to Spain temporarily, got married, and submitted his dissertation in natural sciences entitled “Acerca de los mamíferos de Filipinas” [Regarding the Mammals of the Philippines]. Later, the Spanish Society of Natural History, to which he had been a member since 1883 (Martí de Tortajada and Ruiz Buitrago, 1975), published his dissertation (Sánchez, 1898, 1900). At the end of 1896, he returned to the Philippines and served as a lieutenant in the Guerrilla de Voluntarios de San Miguel. The group was formed in Manila with personnel assigned to the Civil Administration Directorate during the war against the revolutionaries (Sánchez, n.d.).

In 1894, motivated by his interest in anthropology, he enrolled in anatomy at the School of Medicine in Manila, the capital of the Philippines. He completed the first four courses of his career there, until his definitive return to Spain after the loss of the last Spanish colonies (Cuba, Puerto Rico and the Philippines), graduating in Medicine in Madrid in 1900 at the age of 40 (Barras de Aragón, 1949). Since 1899, he was already working in that city, after obtaining, through a competitive examination, the position of second assistant, affiliated with the Zoology section at the Natural History Museum in Madrid (Sánchez, 1899; Sánchez, 2019). In the academic year of 1900–1901, he pursued his doctoral studies in medicine and also took over the chair of zoography of vertebrates due to the illness of the professor and published his work on the histology of snails “Nota sobre el divertículo de la bolsa del *Helix aspersa*” [Note on the diverticulum of the *Helix aspersa* pouch] (Sánchez, 1901).

In 1899, with the aim of working closely with Cajal, he began attending the Laboratory of Normal Histology and Anatomy at the Faculty of Medicine in order to broaden his knowledge of microscopic anatomy (Sánchez, 2019). There, he practiced different histological techniques under the direction of Assistant Professor Eduardo del Río Lara and Cajal himself, as his autobiography records (Sánchez, 2019, p.685):

When Dr. Cajal went up to the laboratory, he used to come up to where I was working, asking me questions about the course of my work. Sometimes he would see some preparations that seemed interesting to us in any way, and he would make certain observations about them that constituted true lessons of eminent wisdom, useful and instructive.

In 1902, he failed in the competitive examinations for the chair of Comparative Anatomy (later called Organography and Animal Physiology) at the Central University (Madrid). Consequently, he was appointed as honorary assistant professor of the Physiology Laboratory at the School of Medicine in Madrid shortly after its foundation, which he held until 1904 (Sánchez, n.d.). In that laboratory he could oversee department practices alongside Manuel Menéndez Potenciano. This role provided him with the opportunity to participate in animal dissections and to expand his knowledge (Sánchez, 2019). Also in 1902, Sánchez submitted his dissertation entitled “Concepto fisiológico de la menstruación” [Physiological Concept of Menstruation] (Sánchez, 1902), which proposed a new theory about the physiological process of menstruation and received an outstanding grade. The dissertation was then partially published in La Correspondencia Médica and La Otorrinolaringología Española (Sánchez, n.d.). Around this time, Cajal offered Sánchez a position as an assistant at the Biological Research Laboratory [Laboratorio de Investigaciones Biológica], founded in 1900. The purpose was to provide Sánchez with “more comfortable working conditions” compared to the Histology Laboratory of medical school (Sánchez, 2019). Thanks to Sánchez’s meticulous work at the Biological Research Laboratory, Cajal honored Sánchez in 1907 by appointing him assistant draftsman of this center (Sánchez, n.d.). Sánchez dedicated “titanic” efforts to improve his drafting skills, with undeniable success (Sánchez, 2019).

In 1907, by a Royal Order, the Junta para la Ampliación de Estudios e Investigaciones Científicas (JAE) [Board for Advanced Studies and Scientific Research] was established, an autonomous public organization presided over by Cajal, and it played a pivotal role in the institutionalization of science in Spain. This institution aimed to connect Spanish culture and science with their European counterparts via research centers, international congresses, and study abroad scholarships. It instituted groundbreaking changes in health and science, making it the most pioneering scientific endeavor for Spain between 1907 and 1939 (Sánchez-Ron, 1988). However, his age, his previous training and his double doctorate in science and medicine, as well as his family circumstances, probably determined that Domingo Sánchez was the only member of the school who was not granted a pension by the JAE to complete his training abroad (González, 1996).

In 1907, the ministerial disposition issued on February 21 authorized the extension of Domingo Sánchez’s micrographic work at the Biological Research Laboratory, under the direction of Cajal. As a result, Sánchez was freed from some of his teaching responsibilities in the faculty to concentrate on his histology research (Sánchez, 2019). In 1910, Sánchez gave up the practice of medicine and the financial benefits that came with it to fully dedicate himself to his laboratory work and emphasize his steadfast dedication to histology and scientific research (Sánchez, 2019).

In the academic year of 1910–1911, he held the position of professor of Organography and Animal Physiology in the absence of the full-time professor. In 1911, Domingo Sánchez joined the Spanish Society of Biology as a founding member, later serving as secretary in 1915. His rise continued, becoming vice-president of the Subcommission on Biology and General Physiology of the National Committee of the International Union of Biological Sciences in 1927. His active participation in this society reflects his influence in the Spanish scientific community (Sánchez, 2019).

In 1920, the Cajal Institute was founded by Royal Decree, bringing together the Biological Research Laboratory and other biological laboratories belonging to the Board for Advanced Studies and Scientific Research. This decision by the Spanish government followed Cajal’s retirement and was made to honor his great prestige. The new building was not inaugurated until 1933 (González, 1996). Initially, Sánchez worked as an assistant draftsman in the Biological Research Laboratory since 1907 until he was promoted to the position of third assistant in the Cajal Institute in July 1926 (Sánchez, 2019). He was deemed indispensable to the center and was authorized by a ministerial decree issued by the Council of Ministers on May 18, 1931, not to be retired. In 1936, Domingo Sánchez was appointed as a retired professor, first assistant, and deputy director of the Cajal Institute (Sánchez, 2019). The Cajal Institute continued to function during the Spanish Civil War (1936–1939) under the leadership of Francisco Tello Muñoz (González, 2005). Sánchez, Tello, and Fernando de Castro were responsible for preventing looting during the siege of Madrid from November 1936 to March 1937 (González, 2005). Following the Spanish Civil War, in 1939, the responsibilities and facilities of the JAE, including the Cajal Institute, were taken over by a new institution called the Spanish National Research Council (CSIC; González, 1996). There he worked until his death in 1947.

## Domingo Sánchez y Sánchez: contributions to neuroscience and histology in Spain

In 1838 and 1839, the botanist Schleiden and the zoologist Schwann independently proposed that the cell is the anatomical and functional unit of all living organisms. Virchow then postulated in *Die Cellularpathologie* (Virchow, 1858) that all cells come from other cells (*ommis cellula ex cellula*), thus configuring what we know today as cell theory. At the end of the 19th century, the nervous system was the exception to this explanation, being the reticular theory proposed by Gerlach (1872), the current theory at the time and supported by most authors, such as Kölliker (1849, 1867) and Golgi (1873) (de Carlos and Borrell, 2007; Sotelo, 2020). According to this theory, the nervous system was formed by a syncytium or diffuse continuous network (*rete nervosa diffusa*) in such a way that the axon of some neurons continued with the dendrites of others. The success of reticularism during its time can be attributed to the fact that the mechanism of communication between neurons via the release of neurotransmitters from the axon terminals had not yet been discovered (Shepherd, 2016). Furthermore, examination of a histologic specimen of stained nervous system cells using light microscopy methods available at the time reveals that the structure of nervous tissue is so intricate that it is seen as a web, and the interpretation of microscopic images depends on the observer (Shepherd, 2016). Soon, researchers such as His (1865) and Forel (1887) began to question these approaches, but it would be Cajal who would finally refute the reticular theory in order to universalise the cellular theory. Although Cajal’s findings initially went unnoticed, they were eventually recognized by important scientists of the time, such as Kölliker (1849, 1867), Retzius (1908), Waldeyer-Hartz (1891), Tanzi (1898), van Gehuchten (1891) and Held (1905) (Ramón and Cajal, 1933; Arias Domínguez, 2018). Finally, the visualization of the space between neural elements was enabled only after the arrival of electron microscopy in the 1950s. This made it possible to confirm one of the central principles of the neural doctrine: presynaptic and postsynaptic elements are separated by a physical space measuring about 10 to 20 nm in width, known as the synaptic cleft (Palade and Palay, 1954; Palay and Palade, 1955; Shepherd, 2016).

In terms of his scientific contribution, from 1902 Domingo Sánchez was the disciple assigned by Cajal to study the histology of the nervous system of invertebrates at the Biological Research Laboratory (Ortiz Picón, 1973). Sánchez adhered to his mentor’s instructions, utilizing Cajal’s modified technique of reduced silver nitrate in his scientific inquiry (Sánchez, 1908). Sánchez’s initial research centered on the snail species *Helix aspersa*, which he had previously studied (Sánchez, 1901). Sánchez tells us about it himself:

Working alongside him, it is not difficult to encounter transcendental questions, the study of which involves solving more or less challenging problems, but of unquestionable importance and interest. He suggests and raises a considerable number of such questions each day and almost always points out paths that facilitate access to the very core of the matters.

It is not surprising, therefore, that guided by such a powerful luminary, I have achieved, in the field of invertebrate neurology, in which I have collaborated with him for so long, several topics of definite interest (Sánchez, 1922a).

**Figure 3:**
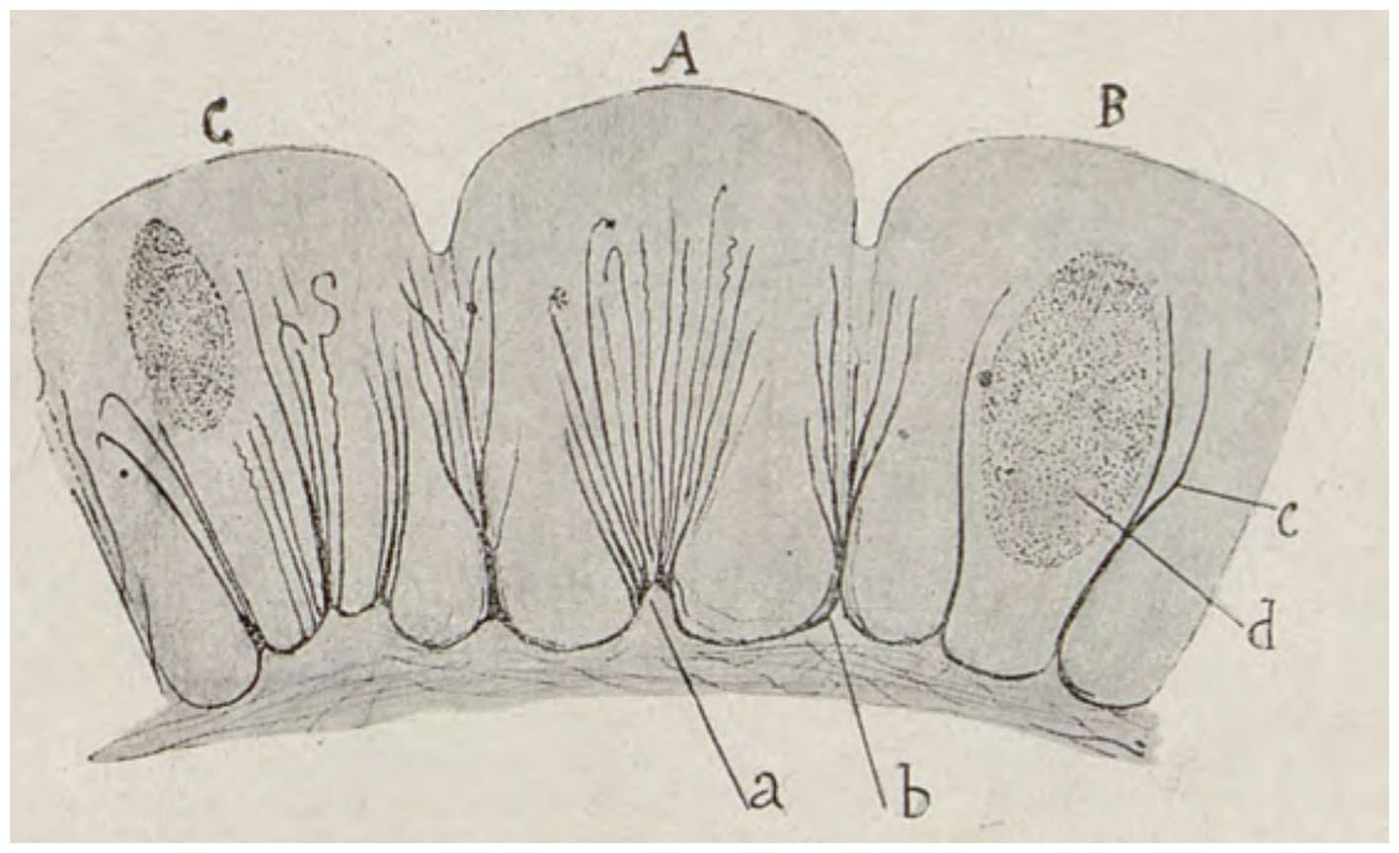
**(A)** In some elements, an infundibulum or cone is evident, from which small ducts diverge through the interior of the cytoplasm, diverging further. **(B)** In other cells, two of these cones can be found, not very short in tubes, which describe curves with concavities facing each other, surrounded by the bulk of the cell nucleus. **(C)** Other cells present several infundibulae. All divergent, but always respecting the structure of the nucleus (Modified from Sánchez, 1904b).

Sánchez’s results yielded outstanding success, and Cajal incorporated some of them into his own work, stating that “the anastomosis supposed by Apathy and Bethe are never seen in such preparations” (Ramón y Cajal, 1903), in order to defend his theory of neuronism against the reticularism. In 1904, Sánchez confirmed the presence of the Golgi apparatus in snails and slugs, which was also discovered by Cajal in *Lumbricus* (Ramón y Cajal, 1904). Sánchez further documented “a network of extremely small ducts located within the intestinal cells of certain isopods” (Sánchez, 1904a,b; Figure 3). Using the optical microscopes available at the time, Cajal and Sánchez described three types of tubular apparatus in cells. One type was like those described by Golgi, while another was a series of drainage or excretory ducts, and the third was a system of nutritive tubes. These discoveries were cited later by various authors (Urtubey, 1931; Levi, 1941). These findings were an initial observation of the Golgi apparatus, the endoplasmic reticulum and the lysosomal system (Martí de Tortajada and Ruiz Buitrago, 1975). One of these drawings of isopods (Sánchez, 1904b), was included in the fourth edition of Cajal’s handbook of Normal Histology and Micrographic Technique (Ramón y Cajal, 1905). This event, as noted by Sánchez himself (Sánchez, 2019), “helped to foster his enthusiasm for research.” Furthermore, in 1907, Sánchez published a paper that equated the reticular structure (Sánchez, 1907), which had been previously described by Cajal in the striated muscles of insects (Ramón y Cajal, 1890), with the discoveries made by the italian anatomist Romeo Fusari in mammals (Fusari, 1894). These observations led to the unification of concepts, and as a result, Sánchez named this structure “Cajal-Fusari,” which was later identified with the sarcoplasmic reticulum (Bowen, 1926). In summary, Sánchez made noteworthy initial observations regarding the Golgi apparatus, lysosomal system, endoplasmic reticulum, and sarcoplasmic reticulum in skeletal muscles, collectively known as the Cajal-Fusari network.

The inclusion of Domingo Sánchez’s research into the work of Santiago Ramón y Cajal exemplified the collaborative dynamic that was the hallmark of the Spanish Neurohistological School. Cajal not only provided his students with guidance but also acknowledged and disseminated Sánchez’s discoveries. Sánchez himself attested to this cooperative approach in his autobiography (Sánchez, 2019, p. 702):

Among the great merits of the Master was that of discovering in other people’s heads cerebral territories that, although apparently sterile, could bear regular and even good fruit if cultivated with assiduity and adequate orientation. Besides, he knew how to guide without violence, almost without the neophyte noticing it, and his guidance was always highly profitable.

At the beginning of 20th century, Bethe (1901), von Apáthy (1907) and Dogiel (1899) gave new wings to reticularism by proposing nerve continuity by means of neurofibrils and joined Golgi’s side who considered that “one single nerve fibre may have connections with an infinite number of nerve cells, as well as with completely different parts of nerve centres which may be a long way from each other”(Golgi, 1906; López Piñero, 2006; Shepherd, 2016). Apháty’s article (1907) questioned and criticized the results of Cajal and his neuronism theory. In response, Ramón y Cajal (1908, pp.24–25) refuted the arguments against his doctrine by referencing Sánchez’s work as justification:

Dr. Sánchez has been tirelessly studying the nervous system structure of hirudineae under our guidance for the past 2 years. His extensive monograph, illustrated with over 60 figures, will soon be published, Aphaty will find an exposition of the main ganglia of leeches with all its details [...]. There, you will see reproduced with all possible accuracy the data obtained both by the Ehrlich and Aphaty procedures and by the reduced silver nitrate method, of which we have modified the formula to obtain more constant and complete results.

Sánchez, 1908 paper, presented at the Zaragoza Congress of the Spanish Association for the Advancement of Science, demonstrated the high level of dedication and thoroughness in his work with reduced silver nitrate. He argued that this method was not limited to studying the nervous system, as it was believed, but should be considered one of the best methods for differentiation, especially for identifying the topographical distribution of tissues and organs (Sánchez, 1908).

**Figure 4:**
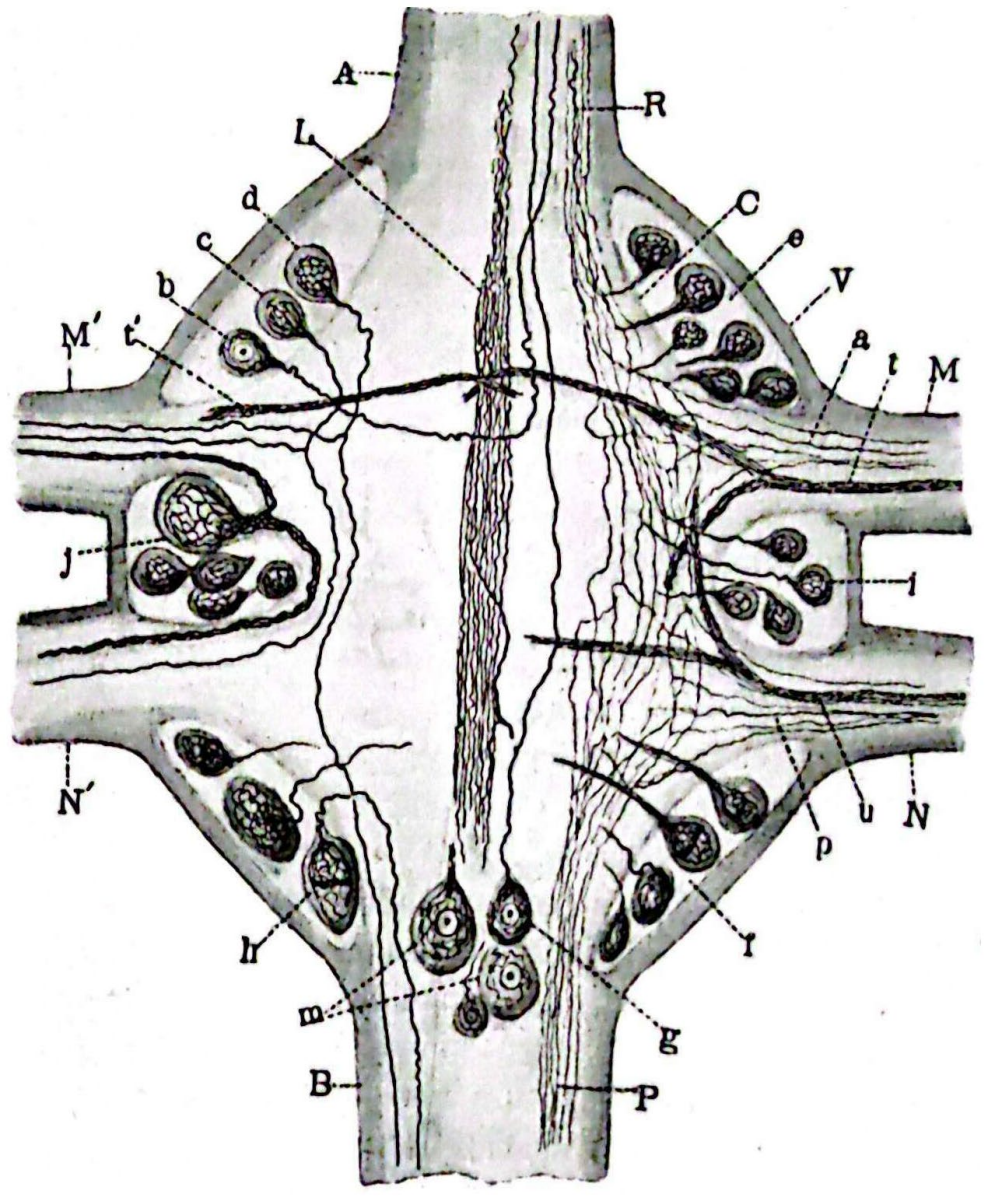
Horizontal section of a median ganglion of *Haemopis sanguisuga*. **(V)** External capsule; **(C)**, Internal idem; **(A)**, Anterior longitudinal cord; **(B)**, Posterior idem; **(M,M’)** Anterior nerves with independent fiber **(A)** and bundles **(T,T’)** passing through them; **(N,N′)** Posterior idem with fibers **(P)** and bundles **(U)** running through them; **(B–D)** Cells of the left anterio-lateral focus; **(E)**, Idem of the right; **(I,J)** Idem of the medial or inter-radicular laterals; **(F)** Idem of the right postero-lateral; Idem of the left; **(G,M)** Idem of the posterior central; **(L)** Odd longitudinal bundle; **(R,P)** Anterior and posterior coordinate fibers (Sánchez, 1912).

As advanced by Ramón y Cajal (1908), Sánchez conducted famous studies (Sánchez, 1909; Sánchez, 1911) on the nervous system of hirudineae (leeches; Figure 4). These studies were renowned for their thoroughness and provided detailed descriptions using various staining methods, of the structure of the abdominal ganglionic chain and peripheral nerves of leeches, including shape, arrangement, and route. The works presented include descriptive and graphic details supporting the structure of nerve cells and their reciprocal relationships, as proposed in Cajal’s neuronal theory. Additionally, Sánchez studied muscle fibers in invertebrates and the motor nerve endings in insects, finding similarities to vertebrates (Sánchez, 1913a, b).

Sánchez dedicated several years to extensively studying the retina and nerve centers of insects, publishing a plethora of papers between 1916 and 1921 under his name (Sánchez, 1916a, 1918, 1919, 1920, 1921). Perhaps the most significant contribution to his career was the work he co-authored with Cajal (Ramón y Cajal and Sánchez, 1915), which vividly displays the mutual fascination that both scientists had for the histology of insects (p1):

Surprisingly little attention has been given to the nervous system of arthropods, especially insects. These creatures possess an incredibly intricate and differentiated nervous system, with a structural delicacy that approaches the limits of the ultra-microscopic. [...] In constructing its impressive creations, the wonder of life always shines more brightly in small things than in the large.

**Figure 5:**
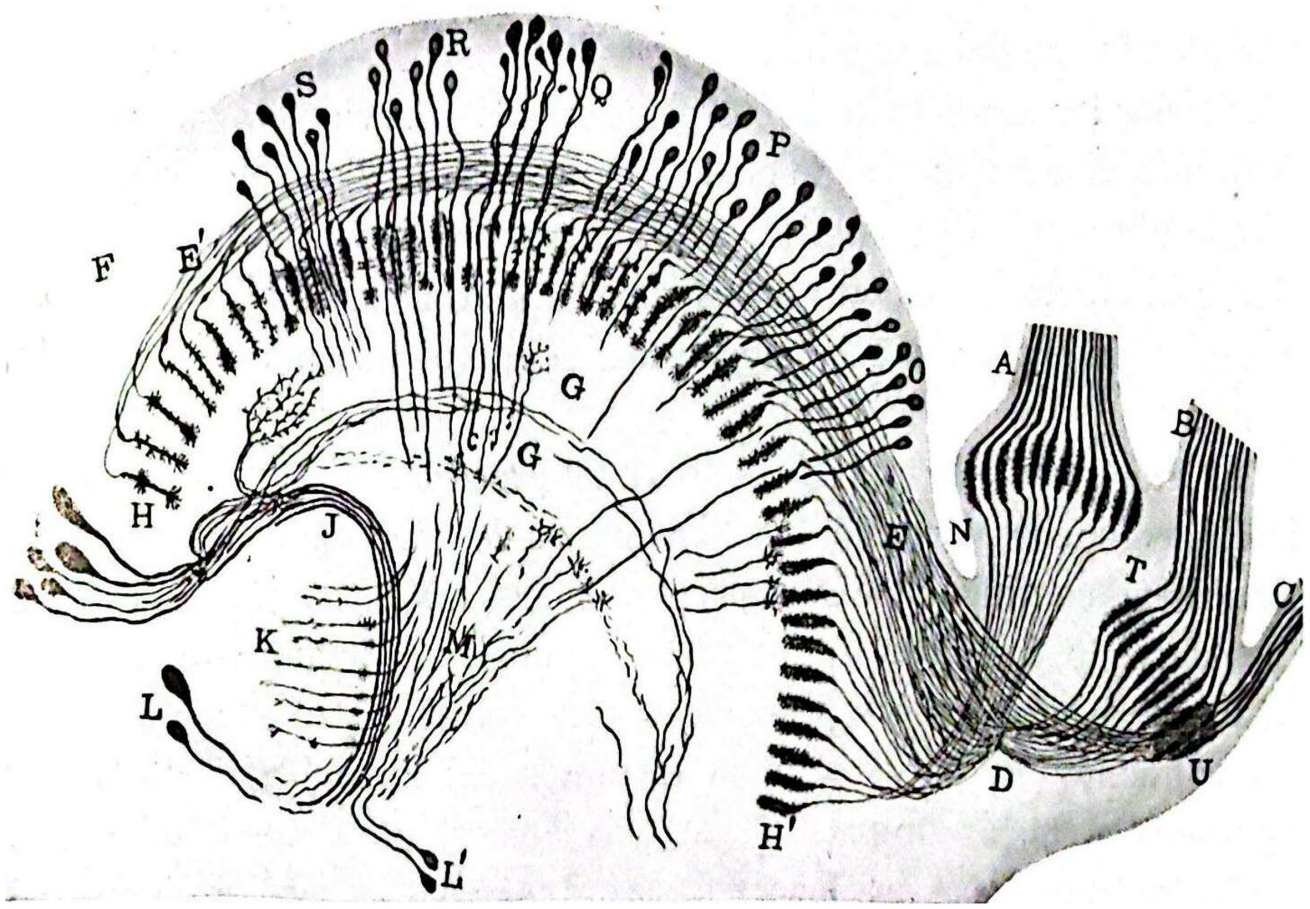
Slightly schematized set comprising the deep portions (ganglionic lamina of the perioptic, intermediary chiasm, deep retina or epioptic, inner chiasm and optic lobe) of the visual apparatus of a chrysalis of *Pieris brassicae* caught in early March. The nerve bundles are identified by capital letters (1917; Sánchez, 1922a).

**Figure 6:**
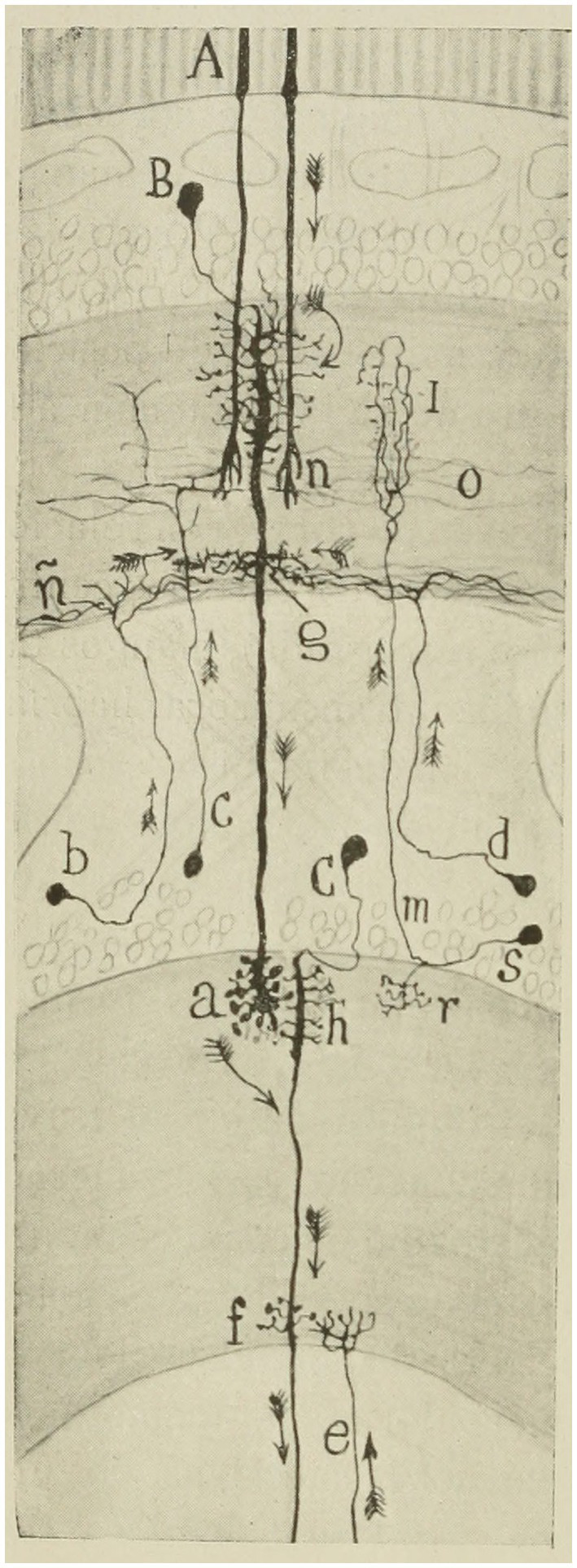
Scheme intended to show the probable course of currents in the bee retina. **(A)** rods; **(B)** second visual neuron (giant monopolar); **(C)** ganglionic corpuscle (third visual neuron); **(B,C)** short centrifuges; **(M,S)** T cell or interzonal connection cell; **(F,G)** basal dendrites destined to articulate with centrifugal fibers (Ramón y Cajal and Sánchez, 1915).

**Figure 7:**
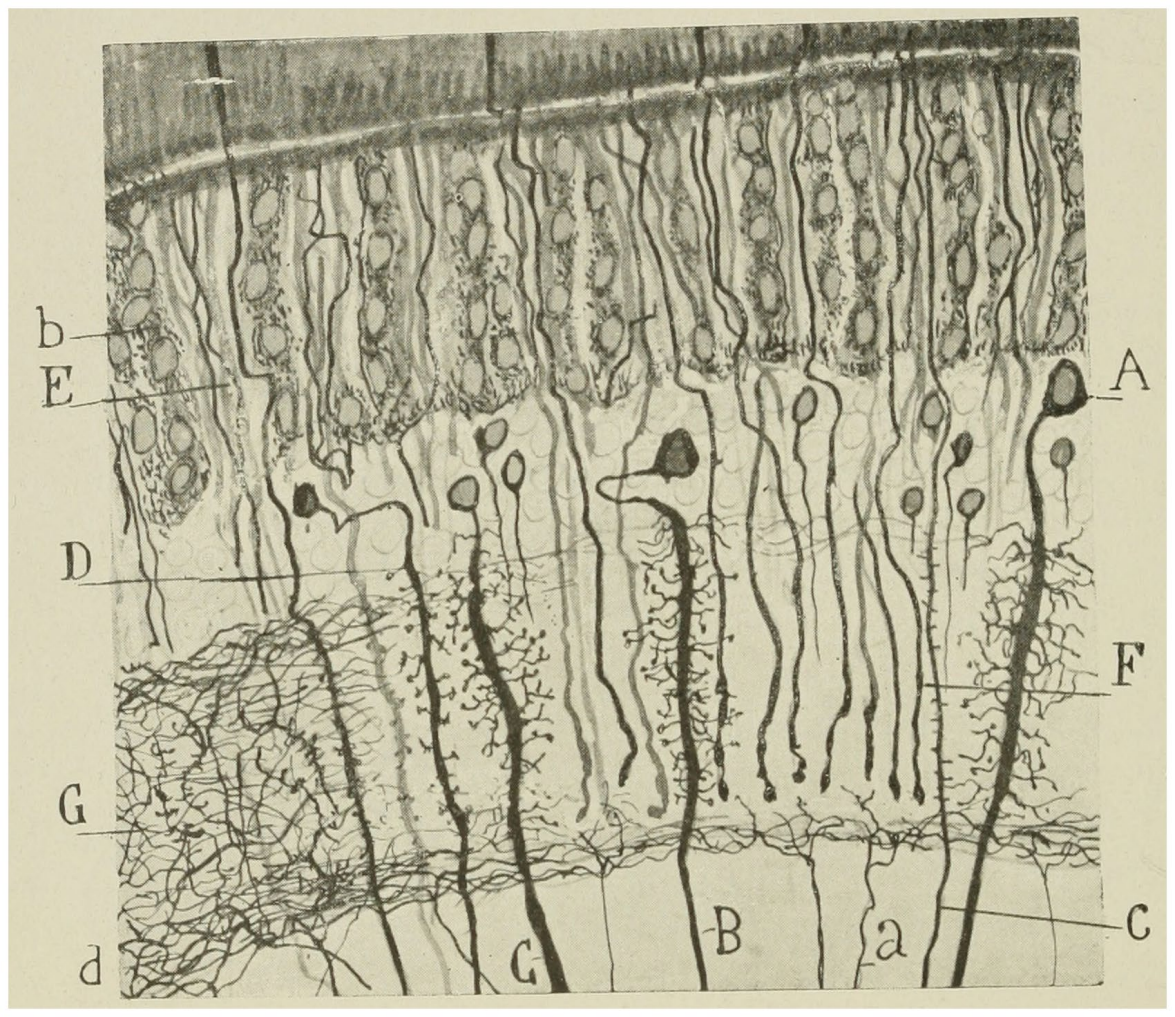
Lepidopteran (*Sphinx*) intermediate retina. **(A–C)** large monopolar cell types; **(D)** small monopolar; **(E)** rod bundle; **(G)** nerve plexus of the external plexiform zone; **(B)** pigment cells; **(D)** inferior limiting nerve plexus; **(C)** rod or long visual fiber (Ramón y Cajal and Sánchez, 1915).

**Figure 8:**
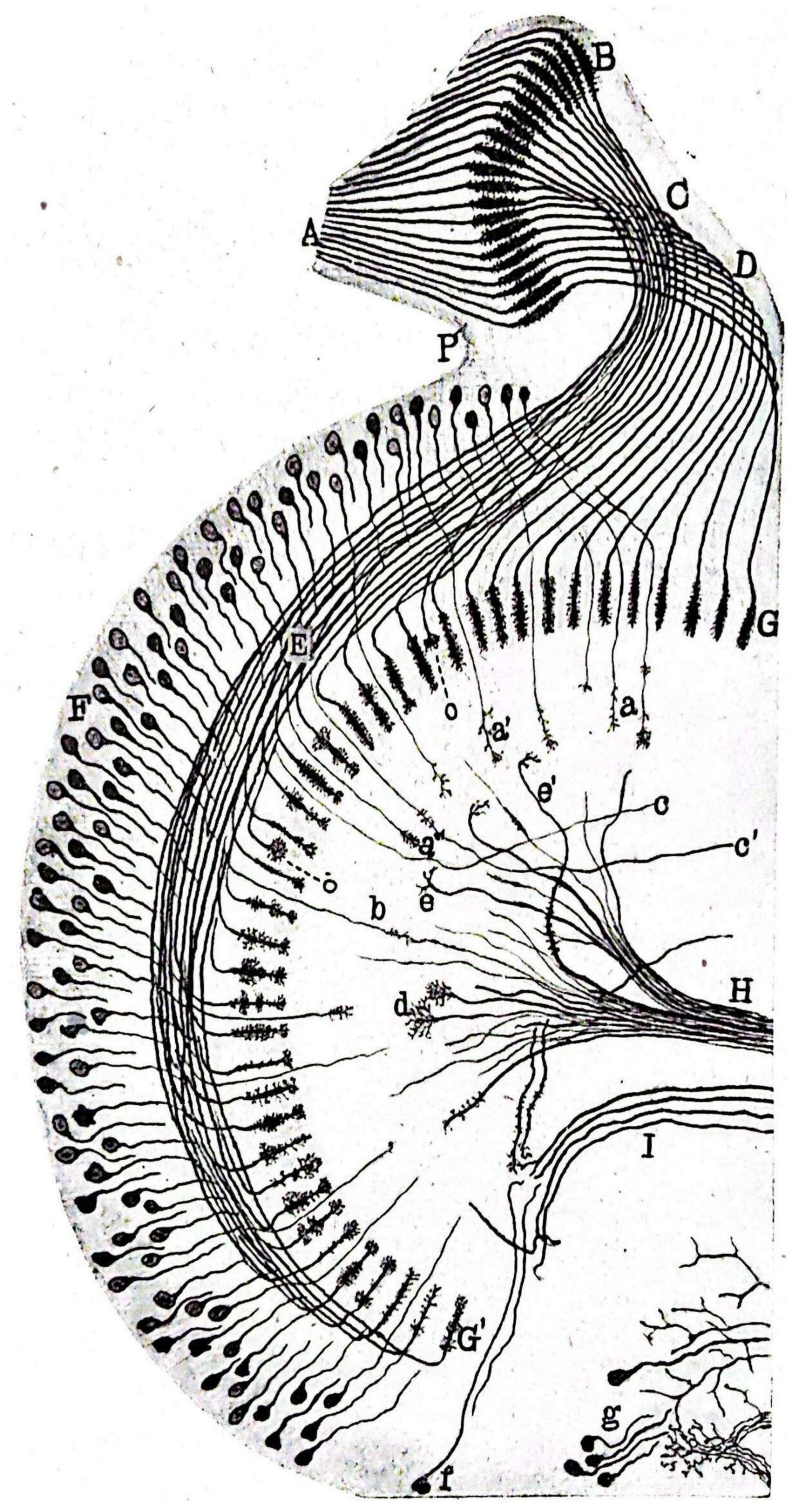
Schematic representation of a horizontal section of the intermediate and inner retinae of a *Pieris brassicae* chrysalis caught in March. **(A)** columella or nerve cord of the fenestrated zone as it reaches the perioptic generating ganglion mass; **(B)** primitive arborization of long optic fibers in the still rudimentary intermediate retina, **(C)** primitive intermediate kiasma; **(D,E)** posterior and anterior groups of long optic fibers extending through the deep retina; **(G**,**G’)** terminal arborizations of these fibers located in the superficial plexus of the inner plexiform mass; **(F)** inner granulosa formation; **(H)** centrifugal fiber bundles forming part of the inner kiasma; **(I)** arciform bundle bordering the ovoid focus of the optic lobe (Ramón y Cajal and Sánchez, 1915).

This study explores the histogenesis and development of retinal and insulating elements found in the peripheral retina of butterflies (Figure 5), alongside the existence of photosensitive cells and the actions of rods and the tactile apparatus within the compound eyes of bees (Figure 6). The study reveals a noteworthy shift in the interpretation of the arrangement of nerve cells in the tactile apparatus due to the high quality of staining achieved for the first time (Sánchez, 2019). In this extensively illustrated work utilizing the silver chromate method, the evolutionary processes of both long and short retinal rods, as well as monopolar and ganglion cells (Figure 7), are extensively documented. Neuronal independence is validated throughout all phases, and the growth cone of nerve fibers is demonstrated to grow and branch (Figure 8). Furthermore, previously unstudied aspects such as the arrangement, origin, and formation of the intermediary chiasm of optic fibers, are described in detail (Sánchez, 1916a). These findings were significant enough that Cajal and Sánchez authored a synthesis of them, which was published in the Boletín de la Real Sociedad Española de Historia Natural volume honoring the fiftieth anniversary of the society (Ramón y Cajal and Sánchez, 1915). Later, Cajal utilized his collaborative work with Sánchez (Ramón y Cajal and Sánchez, 1915) on invertebrate nervous systems to assert his neuronism versus reticularism theory. He “advised those who wish to delve further into this inextricable labyrinth to read the paper he published in collaboration with D. Domingo Sánchez” (Ramón and Cajal, 1933, p. 81).

Sánchez proposed that the compound eyes of insects contain two distinct classes of receptor rods. These rods would correspond to the rods (long) and cones (short) found in vertebrates, enabling insects possessing both receptors to distinguish colors as well as light clarity (Sánchez, 1922b, 1923a). Sánchez identified and drew many types of local interneurons in the fly’s spinal cord (Strausfeld, 2012).

Such was the significance and influence of this collaborative effort with Sánchez that Ramón y Cajal mentioned it in his memoirs (Ramón y Cajal, 1917, pp. 567–569), attesting to its utmost importance:

The second book [...] centered upon the interesting subject of the retina and optical centers of insects. In this work my assistant, D. Domingo Sánchez, collaborated contributing mainly numerous and admirably made preparations. [..] The subject always fascinated me because, to my idea, life never succeeded in constructing a machine so subtilely devised and so perfectly adapted to an end as the visual apparatus. [...] There, in fine, I felt more profoundly than in any other subject of study the shuddering sensation of the unfathomable mystery of life. [...] The complexity of the insect retina is something stupendous, disconcerting, and without precedents in other animals. [...] And I, deceived by the unfortunate preconception of serial progress of zoological structures of similar function, hoped to find a very simple and easily studied structural plan! It is indubitable that zoologists, anatomists and psychologists have lighted the insect. Compared with the retina of these apparently humble e representatives of life (hymenoptera, lepidoptera and neuroptera), and the retina of the bird or higher mammal appears something coarse, rude and deplorably elementary. The comparison of the rude wall clock with an exquisite and diminutive hunting-case watch fails to give an adequate idea of the contrast, for the “hunting-case eye” of the higher insect does not merely consist of more delicate wheels, but contains besides various highly complicated organs which are not represented in the vertebrates.

**Figure 9:**
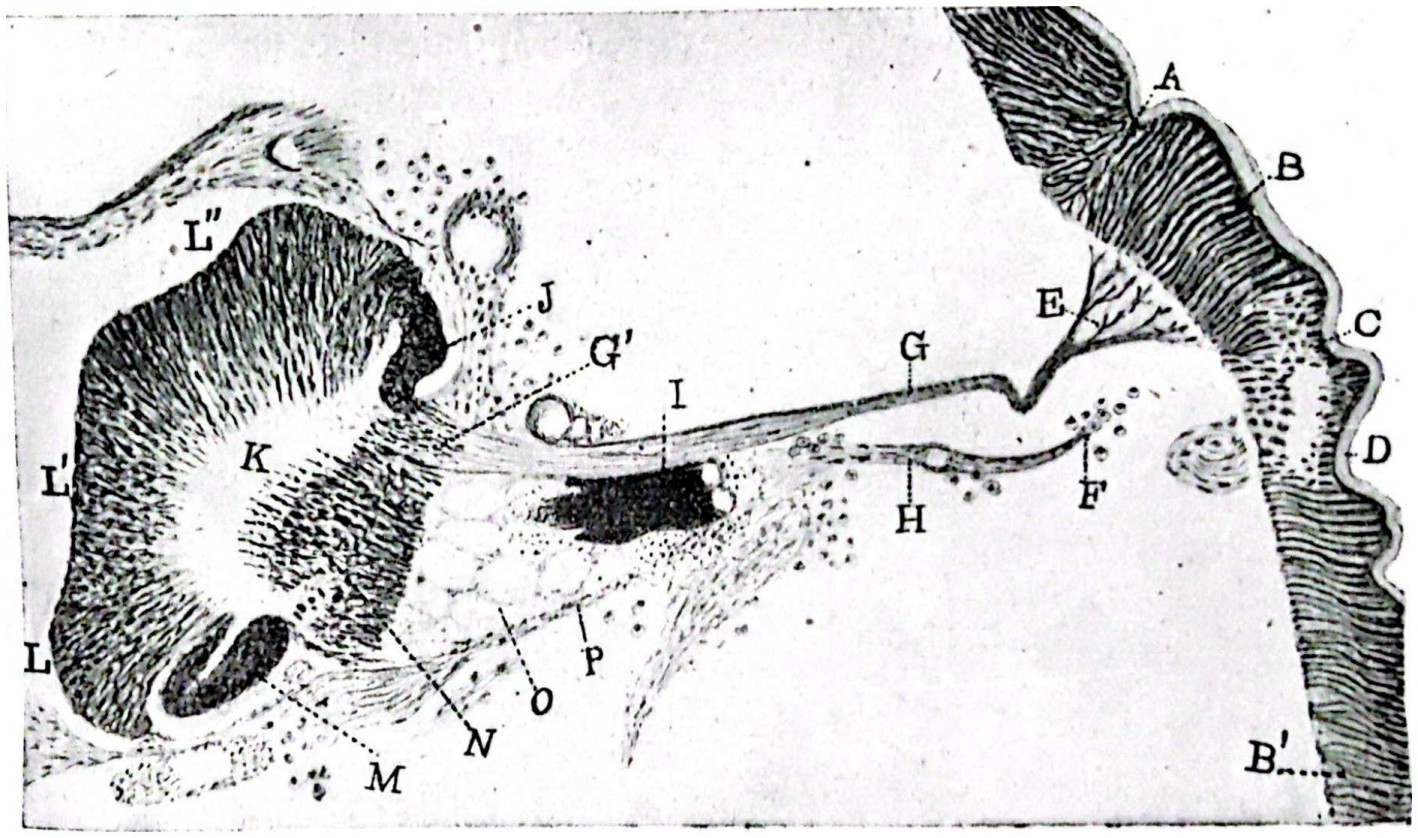
Portion of a nearly horizontal section of the head of a *Pieris brassicae* chrysalis at hatching. Staining with alum hematoxylin (Leutz dib. Prism, obj. 5, tube length 170 mm). **(A–C)** folds corresponding to the limiting furrow of the ocular zone of the hypodermis; **(B)**, ocular hypodermis (future outer retina); **(B’)** general hypodermis of the chrysalis head; **(D)**, chitinous cuticle lining the hypodermis; **(E)**, branching of the columnilla or primitive nerve cord of the fenestrated zone; **(G)** primitive columnilla; **(H)**, remains of the optic nerve of the caterpillar; **(F)** external extremity of the same surrounded by phagocytes; **(I)** group formed by the retinulae and crystallins of two single eyes of the caterpillar; **(G’)** place where the ganglionic layers of the intermediate retina will precipitate to form; **(J)** ganglionic mass that will be incorporated in the part of the intermediate retina; **(K)** inner medullary mass; **(L,L’,L”)** cellular cortex of the celebroid mass; **(M)**, posterior ganglionic mass; **(N)** region of the inner chiasm; **(O)** mass of reticular globes (retinal cells of the caterpillar’s eyes); **(P)** fibrous membrane that formed part of the optic nerve of the caterpillar (neurilemma; Ramón y Cajal and Sánchez, 1915).

**Figure 10:**
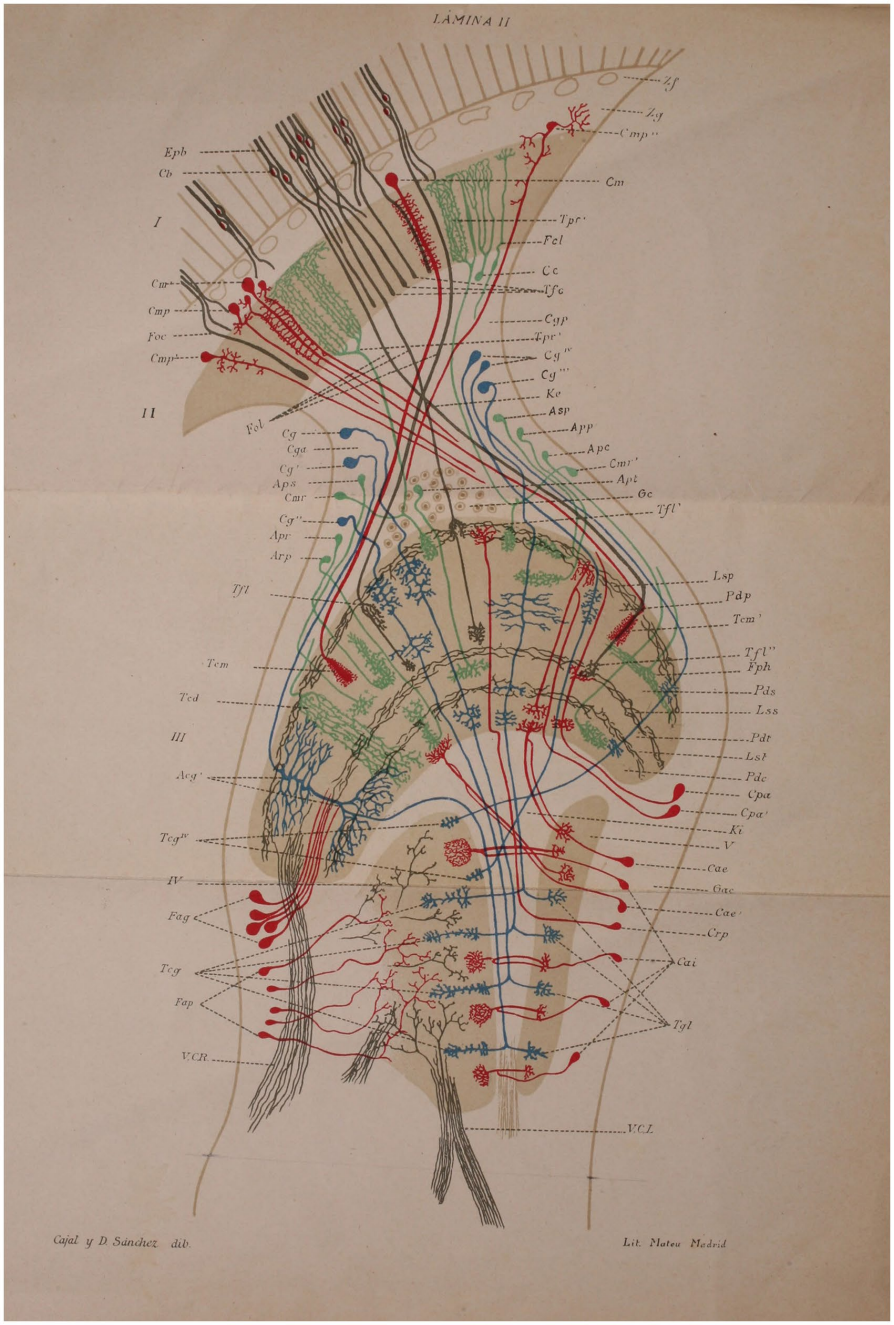
Diagram corresponding to the retina of the bluebottle fly (Ramón y Cajal and Sánchez, 1915).

Building on his previous work, Sánchez continued to study the histolysis of insect nervous centers and its correlation with metamorphosis (Sánchez, 1922a, 1923b, 1924a,b, 1925, 1926a,b; Figure 9). Contrary to the prevailing notion of continuous larval growth to adulthood, Sánchez’s specimens showed degenerative gaps in the nerve ganglia, suggesting reorganization of the nervous system. Furthermore, the coexistence of cells undergoing mitotic division transforms metamorphosis into a multifaceted phenomenon involving histolysis and histogenesis of the nervous system in insect larvae. Sánchez y Sánchez’s studies of optic lobe development are among the first descriptions of programmed cell death, now called “apoptosis,” and he also published important accounts of the postembryonic development of the compound eyes in arthropods (Strausfeld, 2012). The discoveries made were so groundbreaking that Sánchez had spent 2 years reviewing the preparations before publishing his research (Sánchez, 2019; Figure 10). Although there was some initial opposition from Professor Plate of Jena, Germany, Sánchez’s discoveries on the histolysis of insect nervous centers had a significant impact in Europe (Sánchez, 2019). Professor Droogleever Fortuyn from the University of Leiden in the Netherlands, eager to personally verify Sánchez’s data, visited the laboratory from April to May 1924 (Sánchez, 2019). In the same year, Professor Bertil Hanström from Landskrona, Sweden, expressed admiration for Domingo Sánchez’s work on the optical centers of insects and cephalopods in a letter. He showed interest in exchanging publications and staying at the Cajal Institute, but ultimately did not do so, attending instead Professor Fortuyn’s lectures on Domingo Sánchez’s preparations for prestigious neurologists (Sánchez, 2019). These visits and lectures enriched the Spanish Neurohistological School and enhanced its prestige throughout Europe.

During subsequent years, Sánchez devoted himself to studying the cerebroid ganglion of the cockroach *Blata orientalis* and the protocerebrum structure of the worker bee and drone *Apis mellifica* (Sánchez, 1936, 1937a, 1940). Although Kenyon described the antennal nerve as the antennal motor nerve in Kenyon (1896), it was Sánchez (1937a), using reduced silver and Golgi’s impregnations, who demonstrated the basic organization of the vertical lobe, showing sensory terminals to it from the antenna as well as neurons connecting it to the more ventral glomeruli of the antennal lobe (Strausfeld, 2012).

In his later years, he published his research contributions in three parts at Trabajos del Instituto Cajal de Investigaciones Biológicas [Studies at the Cajal Institute of Biological Research]: Tropisms of reflexes and instincts, their genesis and realization (Sánchez, 1943), animal movements and migrations (Sánchez, 1944), and the involvement of the nervous system in instinct realization (Sánchez, 1945). Following Sánchez’s passing, the 37th volume of Papers of the Cajal Institute for Biological Research began with a text by Francisco Tello (1947, p 1), Cajal’s right-hand man, in his honor. Tello’s text gave an overview of Sánchez’s life, work and exemplary character:

[...] His love for science and his iron determination made him overcome the circumstances that hindered his vocation throughout his life and, with exemplary patience and industriousness, he was able to contribute splendidly to the clarification of the structure of the nervous system of invertebrates, expertly handling techniques of great difficulty.

## Domingo Sánchez’s work in other fields

In addition to his work in neurohistology, Domingo Sánchez published several papers on anthropology, paleopathology, criminology, archeology, and ethnology (Sanchez, 1916b; Sánchez, 1923c, 1937b). In 1902, Sánchez secured a position as the curator of the Museum of Natural Sciences of Madrid and assistant professor at the Faculty of Science of the Central University (Madrid), where he was assigned to the chairs of Zoography, Experimental Psychology, and Anthropology. Later he was assigned as assistant professor to the chair of Anthropology while working as curator at the Anthropological Museum. In the old museum of Dr. Velasco, he installed, classified, and ordered many preparations that he himself brought from the Philippines. He continued this work until his retirement in 1931. Additionally, Sánchez taught physics at the Escuela Superior de Artes y Oficios in Madrid. He was appointed numerary professor of extension of geometry trigonometry and topography and elementary physics and electrotechnics in 1905 (Sánchez, 2019). During the academic years 1914–1915 and 1915–1916, Sánchez held the position of professor of Criminal Anthropology at the Spanish Criminological Institute (Sánchez, 2019). In 1921, he co-founded the Spanish Society of Anthropology, Ethnography, and Prehistory along with Francisco Barras de Aragón and Manuel Antón Ferrándiz (Ortiz García and Sánchez Gómez, 1994). He had a great interest in Anthropology, not only publishing in the Journal of the Spanish Society of Anthropology, but also serving as its librarian from 1921 to 1927, as Secretary from 1927 to 1934, and as Honorary Secretary until 1941 (Sánchez, 2019).

His social involvement is noteworthy, as he held the esteemed position of honorary member of the Royal Founding Society of Schools for Orphans and Pensioners of the Teaching Profession in 1909 and became a patron member of the Association for Penitentiary Studies and Rehabilitation of Offenders in 1915 (Sánchez, 2019). At the end of his life, Sánchez was awarded the Couder Prize by the National Academy of Medicine during the session held on March 24, 1944. Additionally, on March 29, he received the plaque of the Civil Order of Alfonso X the Wise (Sánchez, 2019).

## The legacy of Domingo Sánchez y Sánchez

Despite his significant contributions to the science, Domingo Sánchez did not found a school, although he influenced the partial dedication to entomological histology of some members of the school, such as Ortiz Picón (Ortiz Picón, 1993), as well as publishing together with Sánchez and Ramón, 1926, son of Santiago Ramón y Cajal, and his own son Sánchez and Sánchez (1931), whose death prematurely ended the career of a promising scientist (Sánchez, 2019).

Similarly, Sánchez’s work had international impact and influence, evident through his extensive correspondence with distinguished scientists of his time, including J. Turchini, a professor from the Faculty of Medicine in Montpellier, France, and G. Th. Dornesco from Bucharest, Romania; M. Philbert of the Laboratory of Zoology at the Faculty of Sciences in Poitiers, France; J. Havet from the University of Leuven, Belgium; Stefan Koplé, head of the Division of Experimental Morphology at the Government Institute of Agricultural Research in Pulawy, Poland; in addition to Professors Fortuny and Hanström (Sánchez, 2019).

His dedication and talent established him as one of Cajal’s great disciples, as reflected in Ramón y Cajal (1917, p. 578):

In process of formation, and giving promise of abundant fruit, are Arcaute, Fortún, Sacristán, Calandre, Sánchez y Sánchez, Ramón Fañanás, Gil y Gil, Luna, Górriz and others. The list of papers by these investigators is a long one and within the common fervor for the religion on the microscope, each original mind has traveled along a different path. Those named above have been my pupils in the broad sense of the word. All have some part in my life and have shared in my emotions. All have heard me think with haltings words during the absorption of my attention and in the brief parentheses of feverish work.

According to Lorente de Nó (1926) the genuine Cajal’s School was constituted by Domingo Sánchez, Francisco, Fernando de Castro and himself.

## Author contributions

JE-S: Conceptualization, Investigation, Project administration, Resources, Supervision, Writing – original draft, Writing – review & editing. AS-H: Investigation, Resources, Writing – original draft, Writing – review & editing.
